# Shaping resilient flood control system design through net present value assessments

**DOI:** 10.1371/journal.pone.0331225

**Published:** 2025-09-09

**Authors:** Ghazi Al-Rawas, Mohammad Reza Nikoo, Mohammad Reza Hassani, Seyyed Farid Mousavi Janbehsarayi, Hossein Hosseinzadeh Kouhi, Mohammad Hossein Niksokhan

**Affiliations:** 1 Department of Civil and Architectural Engineering, Sultan Qaboos University, Muscat, Oman; 2 Faculty of Environment, University of Tehran, Tehran, Iran; University of Mpumalanga, SOUTH AFRICA

## Abstract

Designing sustainable Flood Control Systems (FCSs) requires considering both the resiliency of the system and the long-term viability of investments. In this regard, our research aimed at integrating concepts of hydrological resiliency and cost-benefit analysis to design the most effective flood control network. To do so, first, the Storm Water Management Model (SWMM) was developed for simulating flood condition. Then, this model was coupled with the Pareto Envelope-based Selection Algorithm-II (PESA-II) to identify the optimal channels’ characteristics and generate a range of non-dominated solutions that balance implementation costs, system resilience (measured by the Simple Urban Flood Resilience Index, SUFRI), and overflow. Different flood management scenarios extracted for North Al-Batinah, Oman, a region under extreme flood events, exhibited high resilience and effectively reduced system overflow with reasonable costs. This highlights the value of optimization in resolving the conflicting objectives inherent in FCS design. Finally, net present values evaluated the long-term economic viability of each management scenario. The results revealed that strategies with moderate design costs and higher SUFRI values yielded optimal financial returns and substantial flood risk reductions. Also, the selected alternative based on net present value could reduce flood volume by 77.9%. This research underscores the critical role of incorporating resilience and cost-benefit analysis into FCS design to enhance the decision-making process.

## 1. Introduction

Flooding in river systems, particularly in developed urban areas, is among the most devastating natural disasters that pose significant risks to communities and can lead to substantial economic damages and human losses [1–5]. Flooding in Oman has repeatedly shown just how vulnerable even developed urban and coastal areas can be. Take June 2007, when Cyclone Gonu dumped nearly 600 mm of rain on Muscat and its suburbs, such as roads and bridges were washed away, hospitals were cut off, and 49 lives were lost, with damages topping US $4 billion [6]. Fast-forward to June 2010, and Cyclone Phet unleashed similar event: at least 24 people died and close to US $780 million in property was destroyed [7]. In May 2018, Category 3 Cyclone Mekunu blew in over Dhofar, dropping rainfall amounts six times the yearly average; risk maps afterward highlighted Salalah’s low-lying districts as hot spots for human and economic exposure [8]. In October 2021’s Cyclone Shaheen overwhelmed Al Batinah with 369 mm of rain in just two days, urban regions were flooded, drainage systems collapsed, and at least 13 people lost their lives [9–10].

The fast growth of cities has created a mix of urban and nonurban catchments, where effective flood management must transcend traditional hydrological boundaries to fully understand the intricate interactions between these regions [11]. An effective way to mitigate flood damage in these areas is to enhance the capacity of flood control channels to make sure they can direct floodwaters to downstream outlets with the least possible risk [12]. To achieve this, innovative performance indicators should be incorporated into the Flood Control System (FCS) design to capture how the system performs throughout the entire flood event. In other words, relying only on traditional design factors such as the number of flooded nodes and inundation volume does not guarantee the reliability of FCS design alternatives. To narrow this, an effective system should also be resilient and able to quickly return to peak performance during flood disturbances [13]. To tackle this, a comprehensive method should be employed to account for socio-economic costs and the timing of investments to ensure FCS plans are sustainable and adaptable for future development [14–17]. In this regard, analyzing flood protection structures requires intersecting the hydrological indices and cost-benefit assessments that link the plans to real-world operational analysis.

Following this hierarchical approach requires using a powerful and flexible rainfall-runoff model to simulate both hydrologic and hydraulic components of integrated urban and non-urban catchments. The Storm Water Management Model (SWMM) [18] is particularly well-suited for this task that offers extensive capabilities, including the ability to code and adjust different component characteristics across numerous model runs [19–22]. For example, research by [11] and [23] demonstrated the SWMM model’s effectiveness in simulating complex urban and non-urban watersheds. By calculating the vulnerable flooded nodes and their overflow volumes, the SWMM model provides designers with a valuable tool for assessing different FCS networks and selecting the most appropriate one based on their objectives. For example, [5] used the SWMM model to assess the operational performance of various FCS design scenarios. Their study analyzed the number of flooded nodes and overflow volumes at vulnerable points to evaluate system effectiveness. While these indicators are useful for evaluating overall system performance, they are based on aggregate data for the entire flood event. However, it is crucial to assess the performance of individual canals and flood control mechanisms throughout the entire flood duration to achieve a more robust flood management strategy. Different studies have been conducted to design the drainage system. For instance, [12] developed frameworks to optimize urban drainage systems, considering different objectives. Their approaches employed optimization algorithms with a focus on traditional objectives, such as efficiency and cost-effectiveness. However, these frameworks often overlooked a crucial aspect: the system’s resiliency. By not including resilience metrics, their methods might overlook how well the system can handle and adapt to extreme weather events and excessive floodwater. Among various indicators, resilience can incorporate the time required for systems to return to full functionality in their analysis [24]. This consideration makes resilience a reliable and innovative concept for flood control network design and analysis. Simple Urban Flood Resiliency Index (SUFRI) developed by [13], is a novel resiliency index that considers different flood indicators, including flooded nodes and volumes. It also introduces a mathematical formula that includes flooding duration and probability of occurrence to offer an effective measure for evaluating FCS performance.

Implementing a sustainable FCS requires balancing the system’s flood control benefits with the execution costs, which incentivizes developers to adopt flood control plans [25–26]. To achieve this, Multi-Objective Optimization Algorithms (MOOA) employs different techniques to search the decision space and find optimal management scenarios. In this context, [27] employed Non-Dominated Sorting Genetic Algorithm-II (NSGA-II) for urban flood management, considering conflicting objectives such as implementation costs and runoff reduction. In applying optimization algorithms, selecting the right objectives can steer the process toward effective solutions and leads to the development of optimal scenarios.

Although MOOAs produce a set of non-dominated plans, but selecting the best one for implementation requires further evaluation. Thus, employing decision-making methods to choose the best solution is critical. As post-analysis steps, these techniques should evaluate flood-management scenarios by quantifying the cost–benefit ratios of each alternative throughout the entire planning cycle. To achieve this, the Net Present Value (NPV) metric can assess the long-term benefits of hydraulic projects over their useful life. This socio-economic indicator allows decision-makers to weigh the costs and benefits of management strategies during the selection process [14,15,28]. Although several studies have explored cost-benefit analysis for various applications, none have applied this approach to select the best scenario from a range of potential solutions in drainage design. For example, [14,15], and [28] researched calculating the NPV for drainage systems. However, their study did not focus on producing optimal scenarios, which in turn limited the decision-making flexibility and the ability to choose the most suitable option from a range of potential alternatives.

This study introduces a novel framework to develop the most resilient flood management strategy. In this regard, we integrated different factors, such as the SUFRI methodology and flood overflow, to optimize drainage system design while considering implementation costs. Also, NPV is applied for the first time to rank optimal flood control network alternatives and identify the most reliable strategy. Moreover, our framwrok links optimal design alternatives with real-world operational strategies, helping identify the most cost-effective and beneficial flood management scenario. Indeed, our study improves adaptability and ensures long-term sustainable performance in mitigating flood risks by addressing multiple dimensions of FCS.

The remainder of this study is organized as follows: Section 2 covers material and methods, including study area information, simulation, optimization processes, and an analysis of resilience and cost-effectiveness of management scenarios. Following this, Section 3 presents lessons learned and results from the research, and finally, Sections 4 and 5 provide the summary and implications, respectively.

## 2. Materials and methods

**Fig 1:**
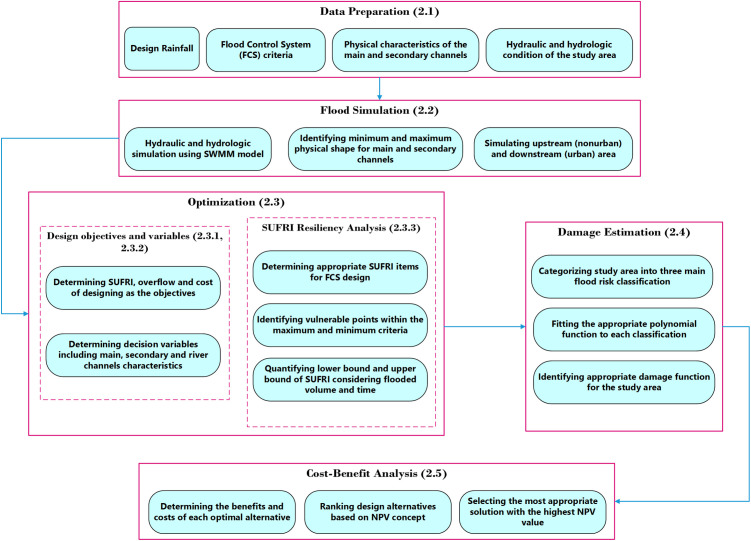
Flowchart of the proposed framework.

This study presents a novel and comprehensive framework for optimizing the design of FCS, incorporating the system’s resiliency from a socio-economic perspective. A brief flowchart of the proposed methodology is shown in **Fig 1**.

### 2.1 Case study and data preparation

**Fig 2:**
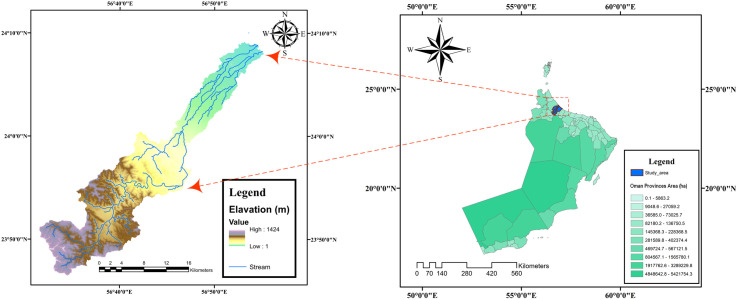
Location of the Wadi Sarami as the case study. (Republished from shapefile dataset provided by the Ministry of Agriculture, Fisheries and Water Resources (MAFWR), Oman, under a CC BY license, with permission from MAFWR, original copyright 2024).

North Al-Batinah, located in northeastern Oman, is an arid region bordered by the Western Hajar Mountains to the west and the Sea of Oman to the north. Its coastal plain is narrowest in the northwest and southeast, expanding to approximately 50 km at its widest point in the center. The plain is composed of alluvial fans that transport sediment from the mountains to the coast. As the second most populous area in Oman after Muscat, North Al-Batinah flat covers more than 90% of the shoreline and has become a hub for human activity. Over the past four decades, concentrated development has driven rapid urbanization and the growth of coastal tourism projects. Major infrastructure, including roads, markets, corniches, fishing harbors, and desalination plants, has been constructed to facilitate coastal living [29]. Also, the flat topography and gentle slope of the North Al-Batinah Coastal Plain result in rapidly expanding flooded areas, even with a minimal increase in flood water height. As a result, the region is vulnerable to flash floods and intense rainfall, as seen during major events like Cyclone Gonu in June 2007, Cyclone Shaheen in October 2021, Cyclone Phet in June 2010, and Cyclone Kyarr in October 2019, all of which led to significant economic and human losses. While areas like the Al-Batinah plain in northern Oman have historically been the most exposed to the impacts of sea-level rise and storm surges [30], an appropriate method or system has not been anticipated for managing storms, which has resulted in damage. This highlights the pressing need to modify planning strategies in the study area to mitigate the risks posed by floods and minimize vulnerabilities for communities, infrastructure, and future development projects. In this study, Wadi Al-Sarami in North Al-Batinah is examined for projecting FCS design (**Fig 2** which is created using ArcMap 10.1).

Hydrological and land use data for Wadi Sarami were sourced from the Ministry of Agriculture, Fisheries, and Water Resources in Oman, to ensure that our model is grounded in accurate, locally relevant data. This dataset included critical watershed characteristics, such as rainfall patterns and land cover, which are essential for realistically simulating flood dynamics in this arid region. The SWMM used in this study is calibrated and validated using two recent high-intensity rainfall events (20 November 2019 and 18 March 2007). The Nash values of 0.97 for calibration and 0.93 for validation demonstrated the excellent performance of the model in flood simulation.

### 2.2 Flood simulation

The Storm Water Management Model (SWMM) was selected for flood simulations due to its widespread use and effectiveness in Hydrological and Hydraulic (H/H) modeling of catchments [18]. The model has proven valuable across diverse study areas with varying characteristics [11,23]. SWMM offers flexibility by allowing detailed representation of watersheds through different sub-catchment levels. The model comprises two main components: the hydrologic module, which processes precipitation to generate runoff, and the hydraulic module, which channels runoff to the catchment’s outfall. SWMM monitors flow quantity in each sub-catchment and channel during simulations. It is also used for designing and sizing drainage systems and can be integrated with various algorithms through coding [18]. This flexibility allows researchers to easily adjust various parameters of the SWMM rainfall-runoff model during the iterative process, such as optimization [31]. To determine design rainfall, we extracted the 1-hour rainfall with a 100-year return period from the intensity-duration-frequency relationships introduced by [32] in the study area. In their research, the Gumbel distribution was fitted to the observed data across 65 meteorological stations in Oman. By doing so, we are able to design a drainage system that can handle rainfall with high intensity and prevent damage to critical infrastructures. Due to the short duration of the simulated rainfall events, we did not incorporate the modules for evaporation, infiltration, and groundwater, nor their associated parameters. Also, the simulated study area is divided into 78 non-urban sub-catchments (upstream) and 209 urban sub-catchments (downstream). The high number of sub-catchments downstream ensures accurate modeling of the urban drainage system, allowing for precise flow tracking through channels and identification of the optimal channel configurations for effective urban flood control.

### 2.3 Optimization of flood control system

Achieving a reliable and sustainable flood control plan requires attention to design objectives and financial constraints. In this regard, application of MOOAs can provide decision-makers with a comprehensive view of how different objectives behave in the decision-making complex space [33]. To better illustrate the optimization process of our study, we have categorized the process into two main layers, which together guide the selection of final optimal alternatives for FCS design:

#### 2.3.1 Layer 1: Defining the design objectives.

The first step in implementing an optimization model is to clearly define the objectives by which the performance of alternatives is evaluated during the selection process. For this study, the three primary objectives are as follows:

1) **Minimizing implementation and maintenance costs**: By focusing on reducing the total costs of constructing and maintaining the flood control system, this objective ensures cost-effectiveness without compromising performance.
2) **Minimizing overflow of the system**: This objective is crucial for ensuring the system’s ability to handle flood events and reducing the likelihood of flooding beyond the drainage capacity.
3) **Maximizing system resiliency under flood events**: This objective tries to consider system resiliency in designing FCS. Traditionally, system performance metrics such as the number of vulnerable points and the total flooded volume were the primary factors in flood management. While these metrics provide useful information, they do not capture the system’s ability to recover during a flood event. In contrast, our study introduces SUFRI as a comprehensive system design metric that reflects the system’s capacity to quickly return to its highest performance during flood disturbance.

In this study, we propose maximizing the minimum SUFRI among all vulnerable points in the system as a novel way of reaching optimal designs. Specifically, for each design configuration (i.e., each combination of channel dimensions), the SUFRI is first calculated at individual vulnerable points across the system. The lowest SUFRI value among these points is then identified, and the optimization process aims to maximize this minimum SUFRI value. This ensures that no point within the system remains critically vulnerable, directing the design towards an overall increase in resilience. By focusing on the minimum SUFRI, the optimization process actively strengthens the weakest points in the system. This approach results in a more uniformly resilient system, where every vulnerable point is reinforced to a certain minimum standard of performance, preventing weak links from compromising the system’s overall efficacy. In summary, the mathematical formulation of the objectives is as follows


Objective1=Minimize(maintanaceandimplementationcosts) (1)

Objective2=Minimize(Overflow) (2)

Objective3=Maximize(MinimumSUFRI) (3)

#### 2.3.2 Layer 2: Determining the decision variables.

The next critical step in the optimization process is defining the decision variables, which directly influence the objective functions. In this study, the primary decision variables revolve around the design of the drainage channels, with two options for adjustment: widening and deepening the channels. These modifications affect the system’s capacity to handle flood volumes and directly impact the performance in terms of cost, overflow, and resilience.

To ensure efficient flood management, three types of channels, Secondary, Main, and River channels have been incorporated into the system design. Each channel type plays a specific role in transferring floodwaters safely to the outlet and forming an interconnected network that maximizes flood control.

- **Secondary channels**: These channels collect floodwater from the subcatchments and direct it to the main channels. Their design is crucial for managing localized flooding and ensuring smooth transfer to the larger channels.
- **Main channels:** After receiving floodwaters from the secondary channels, the main channels are responsible for routing these flows towards the river channels. Proper dimensioning of these channels is critical for preventing overflow at this intermediate stage.
- **River channels**: River channels carry floodwater from upstream nonurban areas and, along their route to the downstream outlet, receive additional floodwater from the main channels. In addition to managing the floodwaters entering from upstream, they also collect and direct flows from the downstream main channels to the outlet. This dual role makes them critical for effectively handling floodwaters across the entire study area.

**Fig 3:**
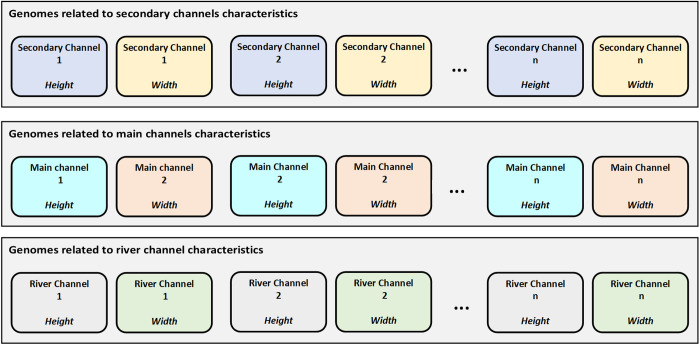
Structure of decision variables for flood control system design.

Given the varying roles and capacities of these channels, the decision variables are categorized into three distinct groups, each tailored to the specific requirements of the channel type. As shown in **Fig 3**, each group of decision variables corresponds to the appropriate channel type, which provides a more targeted approach to system optimization. This categorization ensures that the optimization process can adjust channel dimensions based on their unique roles in managing flood flow.

#### 2.3.3 Resiliency analysis using Simple Urban Flood Resilience Index (SUFRI).

In flood control system design, resilience plays a critical role in determining how well the system can respond to and recover from extreme events. In this study, we apply the Simple Urban Flood Resilience Index (SUFRI) developed by [13] to evaluate the resilience of the designed flood control channels. Building on the concept of urban flood resilience as the ability to maintain or swiftly restore functionality after a flood event [24], SUFRI quantifies this resilience using H/H model outputs. Three key factors are incorporated into the SUFRI calculation:

- **Flood indicator**: Represents the system’s ability to maintain functionality during flood conditions, using indicators such as flood depth and flooded volume.
- **Flood duration**: Measures how quickly the system can return to normal after a flood by considering the length of time floodwaters exceed a critical depth.
- **Flood frequency**: Captures the need for adaptive system changes by quantifying how often flooding occurs beyond acceptable thresholds.

Each of the mentioned components is quantified at vulnerable points within the system and normalized on a scale from 0 to 1, where a value closer to 1 indicates higher resilience. These values are combined into a single SUFRI score (Eq. (4)) for each point to provide a detailed understanding of system performance under flood conditions.


$${SUFRI}_{i}={\left({SI}_{{FloodIndicator}_{i}}{\times SI}_{{Floodduration}_{i}}{\times SI}_{{FloodFrequency}_{i}}\right)}^{\frac{1}{3}}$$ (4)

In this equation, ${SI}_{{FloodIndicator}_{i}}$ represents the flood indicator calculated for the *i*^*th*^ vulnerable point. ${SI}_{{Floodduration}_{i}}$is the term for evaluating the duration of flooding at *i*^*th*^ flooded node. Lastly, ${SI}_{{FloodFrequency}_{i}}$calculates how often the *i*^*th*^ point exceeds acceptable threshold. The normalization of each component is achieved using the following logistic function:


$${SI}_{i}=\frac{1}{1+e^{\alpha (x_{i}-x_{o})}}$$ (5)

Where the α is the weight assigned to the SUFRI element, $x_{i}$ represents the statistic of interest (flood indicator, duration, or frequency) at location *i*, and $x_{o}$ is the standard or threshold value for the SUFRI element. To narrow this, in logistic equation of Eq. (5), α is the steepness parameter that controls how quickly the transition occurs between the lower and upper bounds of the logistic curve. In this study, the flood indicator is determined as the flooded volume to better set the channel capacity required for safely transferring floodwater. Also, as we evaluate the performance of the flood control network based on the event-based approach, the flood frequency for all the vulnerable points is set to 1. It should be noted that the system’s resilience is equal to the average of SUFRI for all vulnerable points.

### 2.4 Damage estimation

To effectively assess flood damage in a specific area, a clear relationship is needed to guide further analysis that identifies an alternative that best meets our needs for reducing flood damage. The damage percentage is defined as the ratio of repair costs to the building’s pre-event market value [34]. Direct physical damage is limited to a maximum value per object, and damage functions are utilized to estimate the fraction of this maximum damage based on the flood depth. Several factors influence flood-related deaths and injuries, including flood characteristics, land use, and the inherent vulnerability of a specific region [35]. To establish a relationship between flood depths and flood damages in different land uses, a polynomial function can be fitted to the depth-damage curves [36]. In this regard, [37] performed a comprehensive analytical and empirical study, offering flood depth-damage data for residential properties, transportation systems, infrastructure, and other sectors across different countries across continents. While depth-damage curves are ideally used for the specific case study where the data were collected, it is common research practice to extrapolate them to similar regions [38–40]. Here, we try to develop three polynomial functions to fit data presented by [37], representing damage to transportation, residential buildings, roads, and other infrastructure.

### 2.5 Cost-benefit analysis

After identifying the optimal flood management scenarios for the drainage system, a method is needed to analyze operations and select a viable alternative. This approach must utilize the cost-benefit analysis to enhance the decision-making process by evaluating the financial flexibility of flood management options. Also, this method should enable us to identify the most efficient alternative through an investment analysis, thereby maximizing economic benefits. To tackle this challenge, in this study, Net Present Value (NPV) is employed to evaluate the benefits of optimal flood management scenarios. This method evaluates flood control measures by discounting their annual costs and benefits throughout the planning cycle, offering a clear picture of their long-term value. The NPV value of the management scenario *i* can be calculated from Eq. (6).


$${NPV}_{i}=\sum_{t=0}^{T}\frac{\left(B_{t,i}+C_{t,i}\right)}{(1+r{)}^{t}}\mathrm{\backslash ]}$$ (6)

Where $B_{t,i}$ and $C_{t,i}$ denote the revenue of the flood control and cost of the *i*^*th*^ management scenario in year t, respectively. Additionally, *r* represents the discount rate, and *T* is the total duration of the investment project. Here, the benefit of implementing flood management scenarios $B_{t,i}$ (in Eq. (6)) represents the reduction in flood damage achieved under the *i*^*th*^ management scenario compared to the flood damage that would occur without control measures. Methodologically, hydraulic projects like this one should have a useful life of 30 years (T = 30), including both the construction and operational phases [14]. It is important to note that, after the initial three years allocated for constructing the main project, the costs transition to maintenance costs for the project for the remaining 27 years. In other words, the execution of the works is estimated to take three years. In this context, we examined how an optimal management scenario for drainage design can be advantageous under a specific rainfall event for 30 years.

## 3. Results

### 3.1 Simulating study area

**Fig 4:**
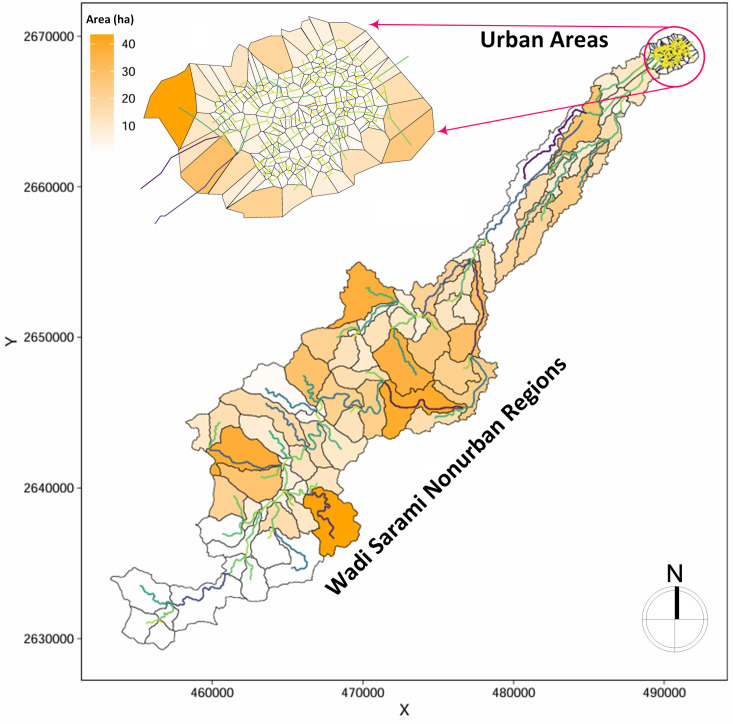
Simulated study area within the SWMM model. Note: Subcatchments are depicted using swmmr package developed by [41].

In the initial step of this research, we conducted a comprehensive H/H simulation of the study area using the SWMM rainfall-runoff model (**Fig 4**). This involved modeling the non-urban upstream areas, covering approximately 230 km², and the densely populated urban downstream regions with about 8.7 km². To enhance the accuracy and flexibility of the design for effective flood management, we classified the urban drainage network into three distinct categories: river channels, main channels, and secondary channels. Each category plays a crucial role in managing water flow from specific catchment areas that helps us to ensure flow is efficiently directed and monitored throughout the system. By employing this strategic categorization, the model allows for precise tracking of flood movement to optimize the size of the flood control network by identifying potential vulnerabilities within the drainage system.

### 3.2 Generating optimal FCS networks

After formulating the H/H conditions of the study area, we now search the multi-objective decision space to identify optimal FCS networks that effectively meet the flood management goals. In this regard, the SWMM-PESAII coupled model was employed to explore and generate optimal combinations of channel characteristics (width and height) that minimize design costs, maximize the SUFRI resilience index, and minimize channel overflow.

In the context of our flood control system optimization, SUFRI was used to evaluate the resilience of vulnerable points within the proposed design. First, the vulnerable points in the system are identified based on the channel configurations produced by the optimization model. At each of these points, the SUFRI is calculated by assessing the flooded volume and duration. Rather than focusing solely on maximizing overall resilience, our novel approach aims to maximize the minimum SUFRI among all vulnerable points. This strategy ensures that even the weakest points in the system are reinforced, preventing any critical failures that could compromise the entire flood management network. By targeting the minimum SUFRI, we guide the optimization process towards designs that provide a uniformly resilient system.

Also, by using SUFRI as a comprehensive resilience metric, we not only address traditional objectives such as cost and overflow management but also improve the system’s ability to handle flood events. The index provides a robust measure of how well the system can function and recover during flood conditions and ensures that resilience is integrated into the design process. This approach enables decision-makers to prioritize solutions that are not only efficient but also sustainable and adaptive in the long term.

**Fig 5:**
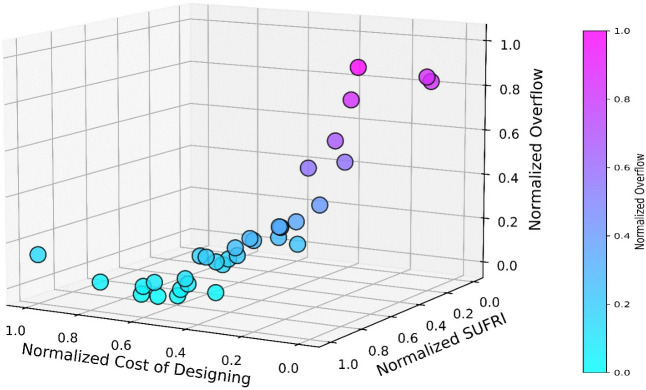
Non-dominated flood management scenarios identified in the multi-objective decision space.

The results of the multi-objective optimization are illustrated in the Pareto front in **Fig 5**. The plot shows the trade-offs between the three key objectives. Each objective is normalized to present on a consistent 0–1 scale for clearer visualization. In this figure, each point represents a unique solution where the three objectives have been evaluated and balanced. A total of 32 non-dominated solutions were generated through the optimization process. These scenarios offer a variety of flood control design options with different cost-benefit trade-offs.

As shown on the horizontal axes, there is a direct relationship between design cost and SUFRI. As the cost of designing increases, the resilience index also increases. This is expected, as a more expensive flood control system is likely to incorporate more advanced and resilient measures, which increases system capacity to withstand floods. However, several cost-effective solutions with low design costs still demonstrate moderately high SUFRI values, which positions them as viable options in terms of both performance and budget.

An interesting pattern emerges between design cost and overflow. As the design cost increases, the overflow generally decreases. The purple-to-blue gradient in the color bar of the figure highlights that high-cost scenarios are associated with less overflow from the channels. The cost-effective scenarios, while more budget-friendly, tend to exhibit higher overflow volumes. The correlation between SUFRI and overflow is quite significant. Solutions with higher SUFRI values tend to have lower overflow, which aligns with the purpose of the SUFRI metric to evaluate both the flooded volume and duration of flooding. This suggests that enhancing the system’s resilience directly contributes to reducing overflow. Also, the highly resilient alternatives on the Pareto front (towards the bottom-left corner of the figure) show minimal overflow, indicating their superior capacity for efficient floodwater management.

As can be observed in **Fig 5**, generally, solutions located on the far right side of the Pareto front, which have a substantially lower cost, indicate maximum overflow and low SUFRI values. These extreme solutions represent highly resilient and efficient flood control networks but may be less attractive due to budget constraints. Conversely, the high-cost solutions, clustered in the bottom-left corner of the figure, show low overflow and higher resilience, potentially showing how more cost can significantly increase the system’s resiliency. Decision-makers must balance between minimizing costs and improving the system’s resilience and ability to manage overflow. In this regard, in the next sections, a novel metric is utilized to select the most appropriate scenario for implementation.

### 3.3 Damage curves

Following the simulation of the study area, it is essential to evaluate the impact of implementing FCS optimal scenarios on flood damage reduction. In this regard, after fitting polynomial relationships to the data provided by [37], three fourth-degree formulas were developed. Each formula represents a specific land use type (transportation, residential buildings, and infrastructure), estimating potential damage during a flood event. Eqs. (7) to (9) denote the relationships between flood depth and damage ratio.


$${Damage}_{Transport}=-0.00099472\times d^{4}+0.02279933\times d^{+}-0.19815414\times d^{2}+\text{0}.\text{73721341}\times \text{d}+\text{0}.\text{0118616}$$ (7)

$${Damage}_{Infrastructures}=-0.00230344\times d^{4}+0.0323194\times d^{3}-\times d^{2}+0.17819863\times \text{0}.\text{569883074d}+\text{0}.\text{0012923}$$ (8)

$${Damage}_{ResidentialBuildings}=-0.00217266\times d^{4}+0.03420206\times d^{3}-0.29030477\times d^{2}+0.6575203\times \text{d}+\text{0}.\text{015350}$$ (9)

**Fig 6:**
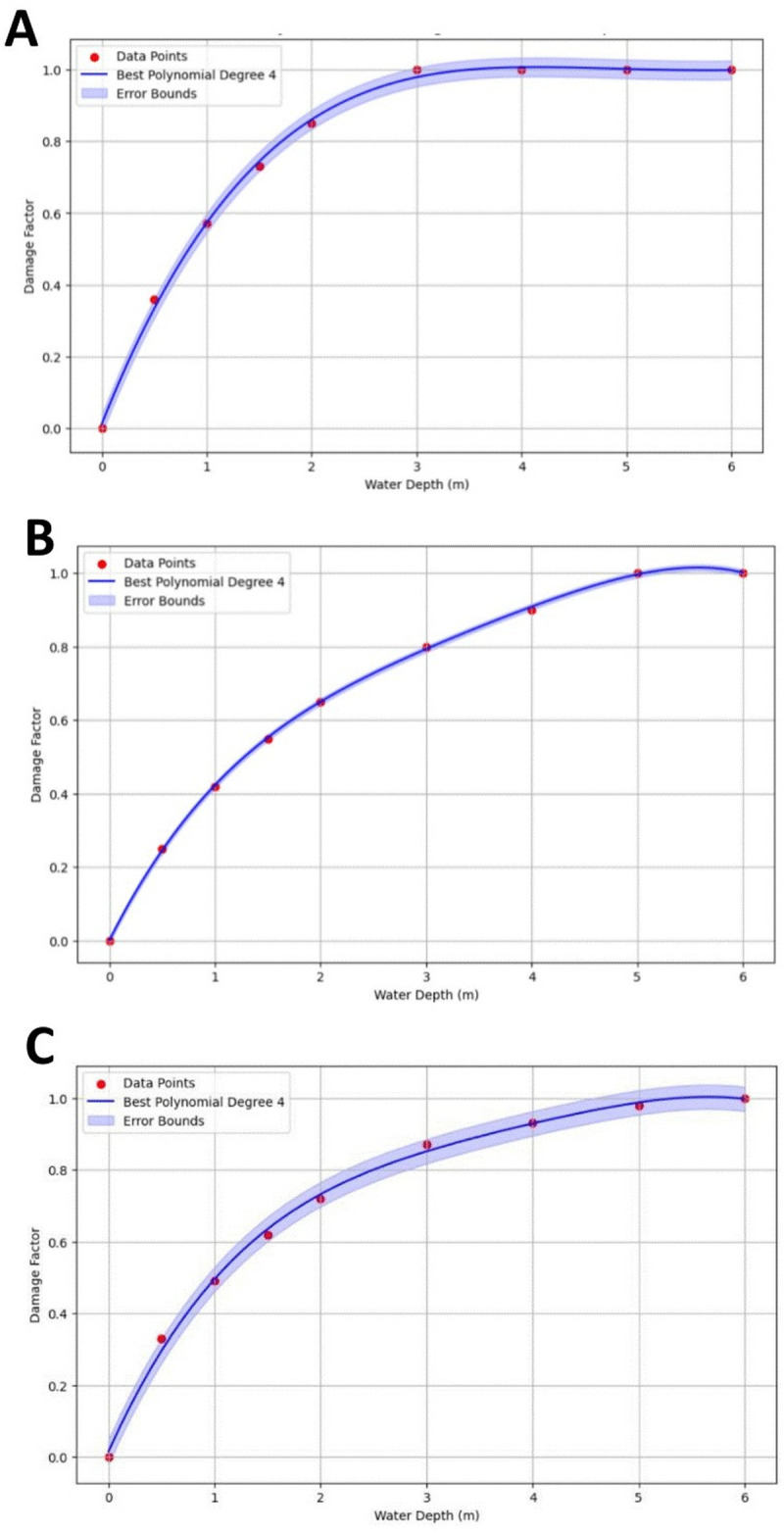
Flood depth-damage curves for A) Transportation, B) Infrastructure, and C) Residential buildings.

Where *D*_*i*_ represents the flood damage ratio, and *d* is the flood depth (in meters). To demonstrate the accuracy and performance of the fitted polynomial curves, **Table 1** presents a detailed comparison of the modeled data using five error metrics: Mean Absolute Error (MAE), Root Mean Square Error (RMSE), R-squared (R^2^), and Error Margin. Additionally, **Fig 6A–6C** visually illustrates the damage curves corresponding to each relationship, which clearly shows the correlation between flood depth and damage to transportation, infrastructure, and residential buildings, respectively. The results demonstrate that the damage curves are accurately fitted to the data and show a high level of precision in capturing the relationship between flood depth and damage. This effective fit is further supported by the strong performance metrics, such as the low R^2^, MAE, and RMSE values. These indicators confirm that the polynomial functions successfully model the flood damage across various land use types in the study area.

**Table 1:** Accuracy and reliability of the fitted curves.

Error factors	Transportation	Residential buildings	Infrastructures
**MAE**	0.0108	0.0124	0.0035
**RMSE**	0.0135	0.0157	0.0044
**R** ^**2**^	0.9983	0.9976	0.9998
**Error Margin**	0.0262	0.0341	0.0082

### 3.4 Investments analysis

**Fig 7:**
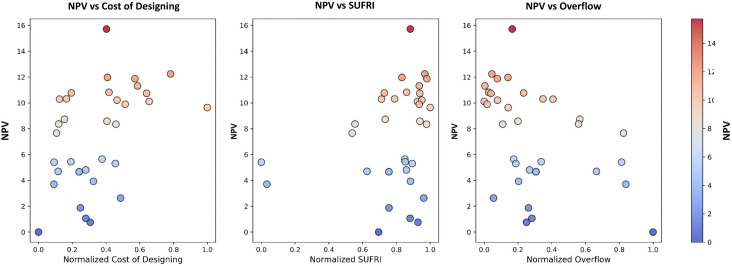
Relationship between NPV and main optimization objectives: cost of designing, SUFRI, and overflow.

After determining the damage curves and calculating the damage ratio for each flooded junction, the next crucial step involves evaluating the cost-benefit analysis of the optimal management scenarios. In this context, the objective value obtained for each point on the Pareto front (benefits) was incorporated into Eq. (6) to determine the NPV for each management scenario. This process enables us to assess the financial viability of the scenarios, considering both the cost-effectiveness and the potential for damage reduction over the project’s lifetime. The three subplots in **Fig 7** visually depict the relationship between the NPV and three key factors of the cost of designing, SUFRI, and overflow beyond drainage capacity. Each point in the graphs represents a potential management alternative on the Pareto front, which is optimized for different objectives. In the first plot (NPV vs Cost of Designing), there is a clear trend where higher NPV values are associated with a relatively moderate cost of designing. A noticeable outlier can be observed in the upper region, where the NPV peaks at approximately 16 while the normalized cost of designing remains between 0.3 and 0.6. This demonstrates that, for specific management alternatives, it is possible to achieve high economic benefits without excessively high design costs. The second plot, NPV vs SUFRI, shows a similar pattern. The higher NPV values are associated with intermediate SUFRI values, typically ranging from 0.4 to 0.8. This indicates that achieving optimal investment benefits does not necessarily require maximizing SUFRI, but rather focusing on scenarios that balance resilience with cost efficiency. The clustering of points in the range of 8–12 NPV suggests a concentrated set of alternatives that offer high benefits under moderate SUFRI levels. The final plot, NPV vs Overflow, shows that lower levels of overflow generally tend to be associated with both low and high NPV values, with a noticeable clustering at the mid-range NPV values (8–12) for overflow rates between 0.2 and 0.6. The presence of outliers, particularly the alternative with an NPV of 16, suggests that some management strategies excel at minimizing economic costs while effectively managing overflow. Conversely, alternatives that fail to manage overflow almost (not strictly) exhibit lower NPV values, indicating a general trade-off between overflow control and investment benefits. Ultimately, these relationships demonstrate that achieving the highest economic benefits in FCS problems requires a balance between design cost, resilience, and overflow control. By doing so, we show the importance of processes of selection that effectively can manage flood risks and evaluate prohibitive costs simultaneously—also considering the adaptability, and real-time operations to improve FCS resilience.

**Table 2** provides a comprehensive comparison between the selected flood management alternative and two Pareto boundary solutions (one with the highest cost and other with the lowest cost). The selected alternative (Pareto point number 17) balances flood control efficiency and economic feasibility. While its flooded volume (7362 m³) is slightly higher than the highest-cost alternative, it offers a significant advantage in terms of NPV (15.71). In contrast, the highest-cost alternative (Pareto point 8) achieves a lower flooded volume of 6945 m³ but at a substantial cost of 16.47 million OMR. Its NPV of 12.24 indicates that the cost-benefit balance is less favorable than the selected solution. Also, the least-cost alternative (Pareto point 31) minimizes implementation costs (15.34 million OMR) but can lead a much higher flooded volume of 10247 m³. Despite the lower cost, its NPV is nearly negligible (0.001), which in turn makes it the least desirable option in terms of long-term financial returns. Also, **Table 2** presents the flooded volume reduction compared to the situation in which FCS is designed based on the lowest boundaries for the size of the drainage system.

**Table 2:** Comparing the selected solution with Pareto boundaries.

	Selected alternative	Alternative with the highest cost	Alternative with the least cost
**Pareto point Number**	17	8	31
**Flooded volume (m**^**3**^)	7362	6945	10247
**SUFRI index**	0.89	1	0.67
**Cost of implementation (million OMR)**	15.92	16.46	15.34
**NPV**	15.71	12.24	0.001
**Flood volume reduction (%)**	77.9	79.2	69.3

## 4. Summary

This study introduced a novel and comprehensive framework for flood control systems (FCS), designed to optimize resilience, cost efficiency, and flood management performance. By employing a hierarchical approach that integrates hydrological simulations, optimization algorithms, and economic analysis, we successfully tackled the multi-dimensional challenge of designing effective flood control systems in urban settings. The framework was built on three key pillars: First, using the SWMM rainfall-runoff model, we accurately modeled both urban and non-urban catchments to track water flow through diverse channel systems. The study area was divided into river channels, main channels, and secondary channels to enhance precision in monitoring and controlling floodwater movement. This detailed simulation provided the foundation for the optimization process by identifying potential bottlenecks and system vulnerabilities. Then, the SWMM-PESAII coupled model was employed to explore multi-objective optimization to balance implementation costs, system resilience (via the SUFRI index), and overflow management. The results demonstrated that it is possible to develop cost-effective flood control strategies that maintain high resilience, as shown by the Pareto front of optimal solutions. These solutions were compared based on their ability to reduce overflow and improve system performance during flood events. The proposed multi-objective optimization generated 32 non-dominated solutions, the specified different trade-offs between cost, resilience, and overflow management. Among these, several cost-effective strategies were identified that demonstrated high resilience (SUFRI values) without excessive costs. These findings showed the value of using optimization techniques to balance the conflicting objectives of FCS problems. Finally, the cost-benefit analysis of the optimal management scenarios was conducted using NPV assessments.

## 5. Conclusion

This approach analyzed the long-term economic viability of each flood control strategy, taking into account both implementation costs and the potential for flood damage reduction. The integration of NPV provided a clear picture of which strategies offered the highest financial returns, with certain cost-effective solutions proving to be resilient and financially beneficial. Results illustrated that higher NPVs were associated with moderate design costs, which proved that optimal strategies can provide substantial financial benefits while effectively managing flood risks. Additionally, resilient systems with higher SUFRI values tended to yield better economic outcomes, which highlighted the importance of incorporating resilience metrics into flood control designs. Findings showed that the selected scenario based on maximum NPV can reduce flood volume by 77.9 percent compared to the scenario with the minimum design criteria while remaining cost-effective for investment.

In conclusion, this study presents a robust and adaptable framework for flood control system design and offers a valuable tool for balancing cost, resilience, and flood management performance. While the findings are promising, addressing the limitations and expanding the framework in future studies will ensure it remains a relevant and powerful tool for flood risk mitigation in an increasingly unpredictable climate. This study opens several avenues for future research: Since the optimal scenarios were generated under the assumption of stationary simulation parameters, future research could focus on evaluating the impact of uncertainty on the performance of these scenarios. This can be achieved by applying robustness evaluation metrics and bottom-up approaches to assess how changes in key variables affect the outcomes. Additionally, given that even optimally designed scenarios require stakeholder agreement for implementation, conflict resolution methods could be explored to integrate the preferences of key stakeholders into the decision-making process. On the other hand, we suggest analysing the cost-effectiveness of nature-based solutions (e.g., green infrastructure, wetlands, and permeable surfaces) as an environmentally friendly method for flood management. Such solutions can complement traditional structural measures and provide additional benefits, including improved water quality, enhanced biodiversity, and increased community resilience. This analysis can help decision-makers determine which method (gray, green, or a combination). better aligns with environmental, social, and flood control objectives to ensure a more holistic and sustainable approach to flood management in future developments.

## References

[1] Halsnæs K, Larsen MAD, Sunding TP, Dømgaard ML. The value of advanced flood models, damage costs and land use data in cost-effective climate change adaptation. Climate Services. 2023;32:100424. doi: 10.1016/j.cliser.2023.100424

[2] Shehzad K. Extreme flood in Pakistan: is Pakistan paying the cost of climate change? A short communication. Sci Total Environ. 2023;880:162973. doi: 10.1016/j.scitotenv.2023.162973 36958563

[3] Dottori F, Mentaschi L, Bianchi A, Alfieri L, Feyen L. Cost-effective adaptation strategies to rising river flood risk in Europe. Nat Clim Chang. 2023;13(2):196–202. doi: 10.1038/s41558-022-01540-0

[4] Hua P, Yang W, Qi X, Jiang S, Xie J, Gu X, et al. Evaluating the effect of urban flooding reduction strategies in response to design rainfall and low impact development. J Clean Prod. 2020;242:118515. doi: 10.1016/j.jclepro.2019.118515

[5] Barkhordari S, Hamze Ghasabsarai M, Garshasbi M, Movahedinia M, Hashemy Shahdany SM. A practical method for rehabilitation of stormwater collecting system by node flooding detection and regional hydraulic redesign: a case study of eastern Tehran metropolis. Water Sci Technol. 2022;86(7):1759–73. doi: 10.2166/wst.2022.312 36240310

[6] Ibrahim OR, Al-Amir M, Al-Maghawry S. Tracking the damages of the Shaheen cyclone in the Sultanate of Oman. Water Pract Technol. 2022;17(12):2548–53. doi: 10.2166/wpt.2022.138

[7] Mansour S. Geospatial modelling of tropical cyclone risks to the southern Oman coasts. Int J Disaster Risk Reduction. 2019;40:101151. doi: 10.1016/j.ijdrr.2019.101151

[8] Al-Awadhi T, Charabi Y, Choudri BS, Bani Oraba Y. Flooding risk analysis: a case study of Muscat Governorate, Sultanate of Oman. Human Ecol Risk Assess. 2017;24(3):667–78. doi: 10.1080/10807039.2017.1396441

[9] Al-Shaqsi S. Care or Cry: Three years from Cyclone Gonu. What have we learnt?. Oman Med J. 2010;25(3):162–7. doi: 10.5001/omj.2010.50 22043331 PMC3191634

[10] Al-Rawas G, Nikoo MR, Janbehsarayi SFM, Hassani MR, Imani S, Niksokhan MH, et al. Near future flash flood prediction in an arid region under climate change. Sci Rep. 2024;14(1):25887. doi: 10.1038/s41598-024-76232-0 39468111 PMC11519630

[11] Hassani MR, Niksokhan MH, Mousavi Janbehsarayi SF, Nikoo MR. Integrated nonurban-urban flood management using multi-objective optimization of LIDs and detention dams based on game theory approach. J Clean Prod. 2024;462:142737. doi: 10.1016/j.jclepro.2024.142737

[12] Lord SA, Ghasabsaraei MH, Movahedinia M, Shahdany SMH, Roozbahani A. Redesign of stormwater collection canal based on flood exceedance probability using the ant colony optimization: study area of eastern Tehran metropolis. Water Sci Technol. 2021;84(4):820–39. doi: 10.2166/wst.2021.273

[13] Hettiarachchi S, Wasko C, Sharma A. Rethinking urban storm water management through resilience – The case for using green infrastructure in our warming world. Cities. 2022;128:103789. doi: 10.1016/j.cities.2022.103789

[14] Ortuño A, Casares J, Calero P, Flor M, Iborra V. A socio-economic and environmental analysis of the implementation of sustainable urban drainage systems in Vega Baja—Alicante (Spain). Water. 2022;14(6):902. doi: 10.3390/w14060902

[15] Gomez-Cunya LA, et al. Analyzing investments in flood protection structures: a real options approach. Int J Disaster Risk Reduction. 2020;43:101377.

[16] Imran M, Sumra K, Mahmood SA, Sajjad SF. Mapping flood vulnerability from socioeconomic classes and GI data: Linking socially resilient policies to geographically sustainable neighborhoods using PLS-SEM. Int J Disaster Risk Reduction. 2019;41:101288. doi: 10.1016/j.ijdrr.2019.101288

[17] Woodward M, Gouldby B, Kapelan Z, Khu S‐T., Townend I. Real options in flood risk management decision making. J Flood Risk Management. 2011;4(4):339–49. doi: 10.1111/j.1753-318x.2011.01119.x

[18] Rossman LA. Storm water management model user’s manual, version 5.0. Cincinnati, OH, USA: National Risk Management Research Laboratory, Office of Research and Development, US Environmental Protection Agency; 2010.

[19] Liu C, Li W, Zhao C, Xie T, Jian S, Wu Q, et al. BK-SWMM flood simulation framework is being proposed for urban storm flood modeling based on uncertainty parameter crowdsourcing data from a single functional region. J Environ Manage. 2023;344:118482. doi: 10.1016/j.jenvman.2023.118482 37413729

[20] Zhu Y, Xu C, Liu Z, Yin D, Jia H, Guan Y. Spatial layout optimization of green infrastructure based on life-cycle multi-objective optimization algorithm and SWMM model. Resour Conserv Recycl. 2023;191:106906. doi: 10.1016/j.resconrec.2023.106906

[21] Assaf MN, Manenti S, Creaco E, Giudicianni C, Tamellini L, Todeschini S. New optimization strategies for SWMM modeling of stormwater quality applications in urban area. J Environ Manage. 2024;361:121244. doi: 10.1016/j.jenvman.2024.121244 38815430

[22] Tanim AH, Smith-Lewis C, Downey ARJ, Imran J, Goharian E. Bayes_Opt-SWMM: A Gaussian process-based Bayesian optimization tool for real-time flood modeling with SWMM. Environ Modell Softw. 2024;179:106122. doi: 10.1016/j.envsoft.2024.106122

[23] Ghodsi SH, Kerachian R, Zahmatkesh Z. A multi-stakeholder framework for urban runoff quality management: application of social choice and bargaining techniques. Sci Total Environ. 2016;550:574–85. doi: 10.1016/j.scitotenv.2016.01.052 26849322

[24] Meerow S, Newell JP, Stults M. Defining urban resilience: a review. Landscape Urban Planning. 2016;147:38–49. doi: 10.1016/j.landurbplan.2015.11.011

[25] Fiorillo D, De Paola F, Ascione G, Giugni M. Drainage systems optimization under climate change scenarios. Water Resour Manage. 2022;37(6–7):2465–82. doi: 10.1007/s11269-022-03187-0

[26] Yu Y, Zhou Y, Guo Z, van Duin B, Zhang W. A new LID spatial allocation optimization system at neighborhood scale: Integrated SWMM with PICEA-g using MATLAB as the platform. Sci Total Environ. 2022;831:154843. doi: 10.1016/j.scitotenv.2022.154843 35351503

[27] Kumar S, Guntu RK, Agarwal A, Villuri VGK, Pasupuleti S, Kaushal DR, et al. Multi-objective optimization for stormwater management by green-roofs and infiltration trenches to reduce urban flooding in central Delhi. J Hydrol. 2022;606:127455. doi: 10.1016/j.jhydrol.2022.127455

[28] Xu K, Zhuang Y, Yan X, Bin L, Shen R. Real options analysis for urban flood mitigation under environmental change. Sustainable Cities Soc. 2023;93:104546. doi: 10.1016/j.scs.2023.104546

[29] Schneider B, Hoffmann G, Reicherter K. Scenario-based tsunami risk assessment using a static flooding approach and high-resolution digital elevation data: an example from Muscat in Oman. Global Planetary Change. 2016;139:183–94. doi: 10.1016/j.gloplacha.2016.02.005

[30] AlRuheili AM. A tale of Shaheen’s cyclone consequences in Al Khaboura City, Oman. Water. 2022;14(3):340.

[31] Mousavi Janbehsarayi SF, Hassani MR, Niksokhan MH, Nikoo MR, Anboohi MS. Multiagent robust decision-making for sustainable stormwater management. J Water Resour Plann Manage. 2024;150(7). doi: 10.1061/jwrmd5.wreng-6410

[32] Chitrakar P, Sana A, Hamood Nasser Almalki S. Regional distribution of intensity–duration–frequency (IDF) relationships in Sultanate of Oman. J King Saud Univ Sci. 2023;35(7):102804. doi: 10.1016/j.jksus.2023.102804

[33] Mousavi Janbehsarayi SF, Niksokhan MH, Hassani MR, Ardestani M. Multi-objective decision-making based on theories of cooperative game and social choice to incentivize implementation of low-impact development practices. J Environ Manage. 2023;330:117243. doi: 10.1016/j.jenvman.2023.117243

[34] Pistrika A, Tsakiris G, Nalbantis I. Flood depth-damage functions for built environment. Environ Process. 2014;1(4):553–72. doi: 10.1007/s40710-014-0038-2

[35] Priest S, et al. Building a model to estimate risk to life for European flood events–Final report. 2007.

[36] Seyedashraf O, Bottacin-Busolin A, Harou JJ. A design framework for considering spatial equity in sustainable urban drainage infrastructure. Sustain Cities Soc. 2022;85:103960. doi: 10.1016/j.scs.2022.103960

[37] Huizinga J, De Moel H, Szewczyk W. Global flood depth-damage functions: methodology and the database with guidelines. JRC105688. Joint Research Centre. 2017.

[38] Banan-Dallalian M, Shokatian-Beiragh M, Golshani A, Abdi A. Use of a Bayesian Network for storm-induced flood risk assessment and effectiveness of ecosystem-based risk reduction measures in coastal areas (Port of Sur, Sultanate of Oman). Ocean Eng. 2023;270:113662. doi: 10.1016/j.oceaneng.2023.113662

[39] Notaro V, De Marchis M, Fontanazza CM, La Loggia G, Puleo V, Freni G. The effect of damage functions on urban flood damage appraisal. Procedia Eng. 2014;70:1251–60. doi: 10.1016/j.proeng.2014.02.138

[40] Apel H, Thieken AH, Merz B, Blöschl G. A probabilistic modelling system for assessing flood risks. Nat Hazards. 2006;38(1–2):79–100. doi: 10.1007/s11069-005-8603-7

[41] Leutnant D, Döring A, Uhl M. swmmr - an R package to interface SWMM. Urban Water J. 2019;16(1):68–76. doi: 10.1080/1573062x.2019.1611889

